# RNA-seq transcriptional profiling of *Leishmania amazonensis* reveals an arginase-dependent gene expression regulation

**DOI:** 10.1371/journal.pntd.0006026

**Published:** 2017-10-27

**Authors:** Juliana Ide Aoki, Sandra Marcia Muxel, Ricardo Andrade Zampieri, Maria Fernanda Laranjeira-Silva, Karl Erik Müller, Audun Helge Nerland, Lucile Maria Floeter-Winter

**Affiliations:** 1 Department of Physiology, Institute of Bioscience, University of Sao Paulo, Sao Paulo, Brazil; 2 Department of Clinical Science, University of Bergen, Bergen, Norway; Institut national de la recherche scientifique, CANADA

## Abstract

**Background:**

*Leishmania* is a protozoan parasite that alternates its life cycle between the sand-fly vector and the mammalian host. This alternation involves environmental changes and leads the parasite to dynamic modifications in morphology, metabolism, cellular signaling and regulation of gene expression to allow for a rapid adaptation to new conditions. The L-arginine pathway in *L*. *amazonensis* is important during the parasite life cycle and interferes in the establishment and maintenance of the infection in mammalian macrophages. Host arginase is an immune-regulatory enzyme that can reduce the production of nitric oxide by activated macrophages, directing the availability of L-arginine to the polyamine pathway, resulting in parasite replication. In this work, we performed transcriptional profiling to identify differentially expressed genes in *L*. *amazonensis* wild-type (*La*-WT) versus *L*. *amazonensis* arginase knockout (*La*-arg^-^) promastigotes and axenic amastigotes.

**Methodology/Principal findings:**

A total of 8253 transcripts were identified in *La*-WT and *La*-arg^-^ promastigotes and axenic amastigotes, about 60% of them codifying hypothetical proteins and 443 novel transcripts, which did not match any previously annotated genes. Our RNA-seq data revealed that 85% of genes were constitutively expressed. The comparison of transcriptome and metabolome data showed lower levels of arginase and higher levels of glutamate-5-kinase in *La*-WT axenic amastigotes compared to promastigotes. The absence of arginase activity in promastigotes increased the levels of pyrroline 5-carboxylate reductase, but decreased the levels of arginosuccinate synthase, pyrroline 5-carboxylate dehydrogenase, acetylornithine deacetylase and spermidine synthase transcripts levels. These observations can explain previous metabolomic data pointing to the increase of L-arginine, citrulline and L-glutamate and reduction of aspartate, proline, ornithine and putrescine. Altogether, these results indicate that arginase activity is important in *Leishmania* gene expression modulation during differentiation and adaptation to environmental changes. Here, we confirmed this hypothesis with the identification of differential gene expression of the enzymes involved in biosynthesis of amino acids, arginine and proline metabolism and arginine biosynthesis.

**Conclusions/Significance:**

All data provided information about the transcriptomic profiling and the expression levels of *La*-WT and *La*-arg^-^ promastigotes and axenic amastigotes. These findings revealed the importance of arginase in parasite survival and differentiation, and indicated the existence of a coordinated response in the absence of arginase activity related to arginine and polyamine pathways.

## Author summary

*Leishmania* are auxotrophic for many essential nutrients, including amino acids. In this way, the parasite needs to uptake the amino acids from the environment. The uptake of amino acids is mediated by amino acid transporters that are unique for *Leishmania*. As part of polyamine pathway, the arginase converts L-arginine to ornithine and furthermore to putrescine, products which are essential for parasite growth. On the other hand, the absence of arginase activity could alter the metabolism of the parasite to surpass the external signals during the life cycle and the fate of infection. The transcriptional profiling of *La*-WT and *La*-arg^-^ promastigotes and axenic amastigotes revealed 8253 transcripts, 60% encoding hypothetical proteins and 443 novel transcripts. In addition, our data revealed that 85% of the genes were constitutively expressed. Among the 15% (1268 genes) of the differentially expressed genes, we identified genes up- and down-regulated comparing the transcript abundance from different life cycle stages of the parasite and in the presence or absence of arginase. We also combined the transcriptional with metabolic profile that revealed a proportional correlation between enzyme and metabolites in the polyamine pathway. The differentiation of promastigotes to amastigotes alters the expression of enzymes from polyamines biosynthesis, which modulates ornithine, L-glutamate, proline and putrescine levels. In addition, the absence of arginase activity increased the levels of L-arginine, citrulline and L-glutamate and decreased the levels of aspartate, proline, ornithine and putrescine in promastigotes by differential modulation of genes involved in its metabolism. Altogether these data provided additional insights into how *Leishmania* is able to modulate its biological functions in the presence or absence of arginase activity to survive during environmental changes.

## Introduction

*Leishmania* is a protozoan parasite that causes widespread human disease known as leishmaniases, characterized by cutaneous, mucosal or visceral manifestations. *Leishmania* alternates its life cycle between the sand-fly vector (promastigote form) and the mammalian host (amastigotes form) [1]. This alternation involves environmental changes and submits the parasite to dynamic modifications in morphology, metabolism, cellular signaling and regulation of gene expression to allow for a rapid adaptation to new conditions. The parasite has also developed resistance mechanisms to evade sand-fly digestive enzymes and the host innate immune response, such as the mammalian complement system and macrophage defense mechanisms involving nitric oxide (NO). NO is produced by nitric oxide synthase 2 (NOS2) using the amino acid L-arginine as substrate [2, 3]. On the other hand, arginase is an immune-regulatory enzyme that can reduce NO production by activated macrophages, limiting the availability of L-arginine to NOS2, supporting *Leishmania* resistance to host defense mechanisms. Arginase uses L-arginine to produce urea and ornithine, a precursor of the polyamine pathway [4]. The success of the *Leishmania* infection depends on the parasite ability to subvert the host defense mechanisms [3, 5]. *Leishmania* also expresses arginase, which supplies the metabolic precursors for parasite replication, an essential step for the establishment of the infection [4, 6].

Our research group has been studying the role of arginase in *L*. *amazonensis* during the parasite life cycle and its role in the establishment and maintenance of the infection in mammalian macrophages [7–9]. Transcriptional profiling has been used in expression studies of several model organisms, including *Leishmania* [10–13]. Holzer et al. (2006) used microarray analysis to determine that 3.5% of the genes were differentially expressed between promastigotes and lesion-derived amastigotes of *L*. *mexicana*, and 0.2% were differentially expressed between promastigotes and axenic amastigotes. The reduced number of regulated genes was a consequence of an increase in the magnitude of the transcript levels in cells under axenic conditions [14]. Leifso et al. (2007) also demonstrated differential gene expression between promastigote and lesion-derived amastigote forms of *L*. *major* [15]. These data indicated that the *Leishmania* genome is mostly constitutively expressed during the parasite life cycle, but there are still some genes that are differentially expressed to adapt to different environmental changes [14–16].

In addition, Goldmann et al. (2007) demonstrated with transcriptome analysis that arginase has an important role in the establishment of infection with *Streptococcus pyogenes*. Arginase type II was up-regulated in the infection of macrophages with *S*. *pyogenes* after 1, 4 and 16 h. However, NOS2 did not show differential gene expression. The same profile was observed in macrophages stimulated with γ-interferon and lipopolysaccharide [17].

In this work, through RNA-seq of *La*-WT and *La*-arg^-^ promastigotes and axenic amastigotes, we identified 8253 transcripts, from which 60% encoding hypothetical proteins and 443 novel transcripts that did not match any previously annotated gene. The transcriptional profiling revealed that 85% of the genes were constitutively expressed. Among the 15% (1268 genes) that were DE, we identified genes up- and down-regulated. Interestingly, we showed 100 genes differentially expressed in *La*-WT promastigotes and 908 genes differentially expressed in *La*-arg^-^ promastigotes. Additionally, we identified 183 genes differentially expressed in *La*-WT axenic amastigotes and only 34 genes differentially expressed in *La*-arg^-^ axenic amastigotes. In summary, our results showed that *L*. *amazonensis* could modulate gene expression with differential regulation between promastigote and axenic amastigotes, indicating that this organism may represent an alternative paradigm for eukaryotic differentiation with minimal contributions from changes in mRNA abundance. The transcriptional profiling also revealed differential gene expression in the development of the *Leishmania* life cycle and the existence of a coordinated response in the absence of arginase activity, providing additional insights into how *Leishmania* is able to modulate its biological functions to survive during environmental changes.

## Methods

### *Leishmania* culture

*Leishmania* (*Leishmania*) *amazonensis* (MHOM/BR/1973/M2269), a strain of our laboratory collection at the Institute of Bioscience, and *L*. *amazonensis* arginase knockout (*La*-arg^-^) [8] promastigotes were grown at 25°C in M199 medium, pH 7.0, supplemented with L-glutamine, 10% heat-inactivated fetal bovine serum, 0.25% hemin, 40 mM NaHCO_3_, 100 μM adenine, 40 mM HEPES, 100 U/mL penicillin and 100 μg/mL streptomycin. Axenic amastigotes of *La*-WT and *La*-arg^-^ were grown in M199 medium supplemented, as described above at 34°C, pH 5.5. For the *La*-arg^-^ cultures, hygromycin (30 μg/mL), puromycin (30 μg/mL) and putrescine (50 μM) were added.

### *In vitro* bone marrow-derived macrophage infection with axenic amastigotes

Bone marrow derived-macrophages (BMDM) were collected from the femur of female BALB/c mice (6–8 weeks) from the Animal Center of the Institute of Bioscience of the University of Sao Paulo. The femurs were washed with cold PBS and the cells were collected at 500 x g for 10 min at 4°C. The lysis of erythrocytes was performed with NH_4_Cl (145 mM) and Tris-base (200 mM) pH 7.0 and incubated on ice for 20 min. After lysis, the cells were washed with cold PBS, collected at 500 x g for 10 min at 4°C and incubated in RPMI 1640 medium supplemented with penicillin (100 U/mL), streptomycin (100 μg/ml), 2-mercaptoethanol (50 μM), L-glutamine (2 mM), sodium pyruvate (1 mM), fetal bovine serum 10% and L929 conditioned medium (15%), as macrophage stimulating factor source. The cells were cultivated for 7 days at 34°C and 5% CO_2_. After differentiation, cellular viability was evaluated with Trypan blue staining 1:1, and cells were counted in a Neubauer chamber.

Approximately 1x10^6^ BMDM were incubated on sterile 13-mm coverslips in 24-well plates overnight at 34°C and 5% CO_2_ to adhere to the coverslips. Non-adherent cells were removed by PBS washing, and the infection was performed with *La*-WT or *La*-arg^-^ axenic amastigotes (MOI 5:1). After 4 h of infection, the cultures were washed with PBS and maintained in culture for 24, 48 and 72 h. Non-infected macrophages maintained in culture at the same conditions were used as control. The infections were evaluated by determining the percentage of infection after counting 200 Panoptic-stained (Laborclin, Parana, Brazil) macrophages. The infection index was determined by multiplying the percentage of infected macrophages by the mean number of parasites per infected cell [18, 19]. Statistical analyses were performed using the t-test.

### Total RNA isolation and library construction

Total RNA from 3 independent biological replicates was isolated from *La*-WT and *La*-arg^-^ promastigotes and axenic amastigotes using TRIzol reagent (Life Technologies, Carlsbad, CA, USA), according to the manufacturer’s instructions. RNA samples were treated with DNase I (Thermo Scientific, Lithuania, EU), and the RNA concentration was determined using a spectrophotometer at A260/A280 (Nanodrop ND1000, Thermo Scientific, USA). In addition, the RNA integrity was evaluated using an Agilent 2100 Bioanalyzer and Pico Agilent RNA 6000 kit (Agilent Technologies, Santa Clara, CA, USA), according to the manufacturer’s instructions. rRNA depletion was performed by poly(A) magnetic beads capture protocol, using Strand-specific TrueSeq RNA-seq Library Prep (Illumina), according to manufacturer´s instruction. Library preparations were performed using Strand-specific TrueSeq RNA-seq Library Prep (Illumina), according to the manufacturer’s instructions.

### RNA-seq and data analysis

Paired-end reads (125 bp) were obtained using the Illumina HiSeq 2000 platform at the Norwegian Sequencing Centre at the University of Oslo. Trimmomatic was used to remove the Illumina adapter sequences [20]. The quality of the produced data was analyzed using FastQC by Phred quality score [21]. Reads with Phred quality scores lower than 20 were discarded. Reads were aligned to the *L*. *mexicana* (MHOMGT2001U1103) genomic data obtained from TriTrypDB version 29 (www.tritrypdb.org) using TopHat (-G option) [22, 23]. Maximum 2 mismatches were allowed. Thereafter, the expression level of the assembled transcriptome and abundance estimation were performed using Cufflinks [24]. The abundance of transcripts was calculated as the Fragments Per Kilobase of transcript per Million mapped reads (FPKM), which reflects the abundance of a transcript in the sample by normalization of the RNA length and the total read number [25]. The gene expression level values were calculated from the transcript counts. Differentially expressed gene analysis was performed on four comparisons pairs (*La*-WT promastigotes vs. *La*-arg^-^ promastigotes; *La*-WT axenic amastigotes vs. *La*-arg^-^ axenic amastigotes, *La*-WT promastigotes vs. *La*-WT axenic amastigotes; *La*-arg^-^ promastigotes vs. *La*-arg^-^ axenic amastigotes). Genes with zero FPKM were excluded (excepted the arginase gene (LmxM.34.1480)). Transcripts that did not match any previous annotated gene were considered novel, and they were identified using Cufflinks with -g option. Statistical significance of DE genes data was determined using independent t-test and fold change in which the null hypothesis was that no difference exists among groups. False discovery rate (FDR) was controlled by adjusting p value using Benjamini-Hochberg algorithm [26]. Functional annotation was performed using GO (Gene Ontology) and the Kyoto Encyclopedia of Genes and Genomes (KEGG). All analyses were performed by Macrogen (www.macrogen.com).

### RT-qPCR validation assays

Reverse transcription was performed using 2 μg of total RNA as a template, reverse transcriptase and random primers (Revertaid H minus Reverse Transcriptase kit, Thermo-Scientific, Canada), according to the manufacturer’s instructions. Equal amounts of cDNA were assessed in triplicate in a total volume of 25 μL containing Power SYBR Green qPCR Master Mix (Life Technologies, Warrington, UK) and the following primers (20 μM): GAPDH_F 5´-TCAAGGTCGGTATCAACGGC-3´, GAPDH_R 5´-TGCACCGTGTCGTACTTCAT-3´, arginase F 5´-TCCTGCACGACCTGAACATC-3´, arginase R 5´-CGCCATGGACACCACCTT-3´, glutamate 5-kinase F 5´-AGCTGGTTTTTGGCGACAAC-3´, glutamate 5-kinase R 5´-CGTCGATGTCGCTGAGAATG-3´, pyrroline 5-carboxylase dehydrogenase F 5´-ACGGTGTTTGTGTATGACGACAGT-3´, pyrroline 5-carboxylase dehydrogenase R 5´-ACCGGTCAGGCCGTACTTC-3´, spermidine synthase F 5´-GCAACCAGGGCGAGTCTATCT-3´, spermidine synthase R 5´-TGACCGTGGAAAAGCCAATAT-3´, amastin-like F 5´-GGAGCGCTACTTCAGCTATGGA-3´, amastin-like R 5´-CGGATCATCAATAAGACGATGTTG-3´, amastin-like F 5´-CGGCTGCCTTTTGCTGTACT-3´ and amastin-like R 5´-CAGACAACGCAAGCTGTGACA-3´. The mixture was incubated at 94°C for 5 min, followed by 40 cycles at 94°C for 30 s, 60°C for 30 s and 72°C for 30 s. A negative control in the absence of reverse transcriptase was included in RT-qPCR assays to detect DNA contamination in RNA samples. The copy number of the target and reference genes were quantified in three biological samples, considering the molar mass concentration, according to a standard curve generated from a ten-fold serial dilution of a quantified PCR product. The normalized *target*/*gapdh* ratio of the molecules absolute number of each target was used as a parameter of the expression. Reactions were carried out using PikoReal 96 RealTime PCR System (Thermo Scientfic, Finland). Analyses were performed using PikoReal Software 2.2 (Thermo Scientific, Finland).

### Ethics statement

The experimental protocols for the animals were approved by the Animal Care and Use Committee from the Institute of Bioscience of the University of Sao Paulo (CEUA 196/2014). This study was carried out in strict accordance with the recommendations in the guide and policies for the care and use of laboratory animals of the São Paulo State (Lei Estadual 11.977, de 25/08/2005) and Brazil government (Lei Federal 11.794, de 08/10/2008).

## Results

### *La*-WT and *La*-arg^-^ axenic amastigotes growth and macrophage infectivity

*La*-WT and *La*-arg^-^ promastigotes in the stationary growth phase were submitted to differentiation in medium, pH 5.5 at 34°C, for 48 h. The growth curve of the differentiated amastigotes of both *La*-WT and *La*-arg^-^ reached stationary growth phase after 10 days of incubation (S1A Fig). Axenic amastigotes were also induced to differentiate back to promastigotes incubating the parasites at pH 7.0 and 26°C that led to differentiation after 48 h of incubation. Moreover, axenic amastigotes infectivity was evaluated. BMDM from BALB/c mice were infected with *La*-WT and *La*-arg^-^ (MOI 5:1), and the infection index was analyzed at 24, 48 and 72 h post infection. According to S1B Fig, both *La*-WT and *La*-arg^-^ axenic amastigotes were able to infect and establish the infection. However, the infection index for *La*-arg^-^ was lower than for *La*-WT, corroborating the previously determined infection index for *La*-arg^-^ promastigotes [8].

Once axenic amastigotes infectivity was confirmed, the total RNA from *La*-WT and *La*-arg^-^ promastigotes and axenic amastigotes was extracted and submitted to RNA-seq, as described in Methods.

### Transcriptomic profiling of *La*-WT and *La*-arg^-^ promastigotes and axenic amastigotes

Transcriptomic analyses were performed using 3 independent biological replicates from each *La*-WT and *La*-arg^-^ promastigotes and axenic amastigotes after Illumina HiSeq2000 sequencing that generated million sequence reads (125 bp) (S1 Table). Sequencing data are available on the NCBI BioProject under accession number PRJNA380128 and Sequence Read Archive (SRA) under accession number SRX2661998 and SRX2661999.

RNA-seq data were aligned to the *L*. *mexicana* genome [27]. After initial assembling, 8253 transcripts and 443 novel transcripts were identified with genome coverage around 90%. And 60% of the transcripts corresponding to hypothetical proteins as listed in S2 Table. The novel transcripts were those that did not correspond to any previous annotated gene, even in *L*. *mexicana* genome, used in the comparisons since *L*. *amazonensis* is not completely annotated.

**Fig 1:**
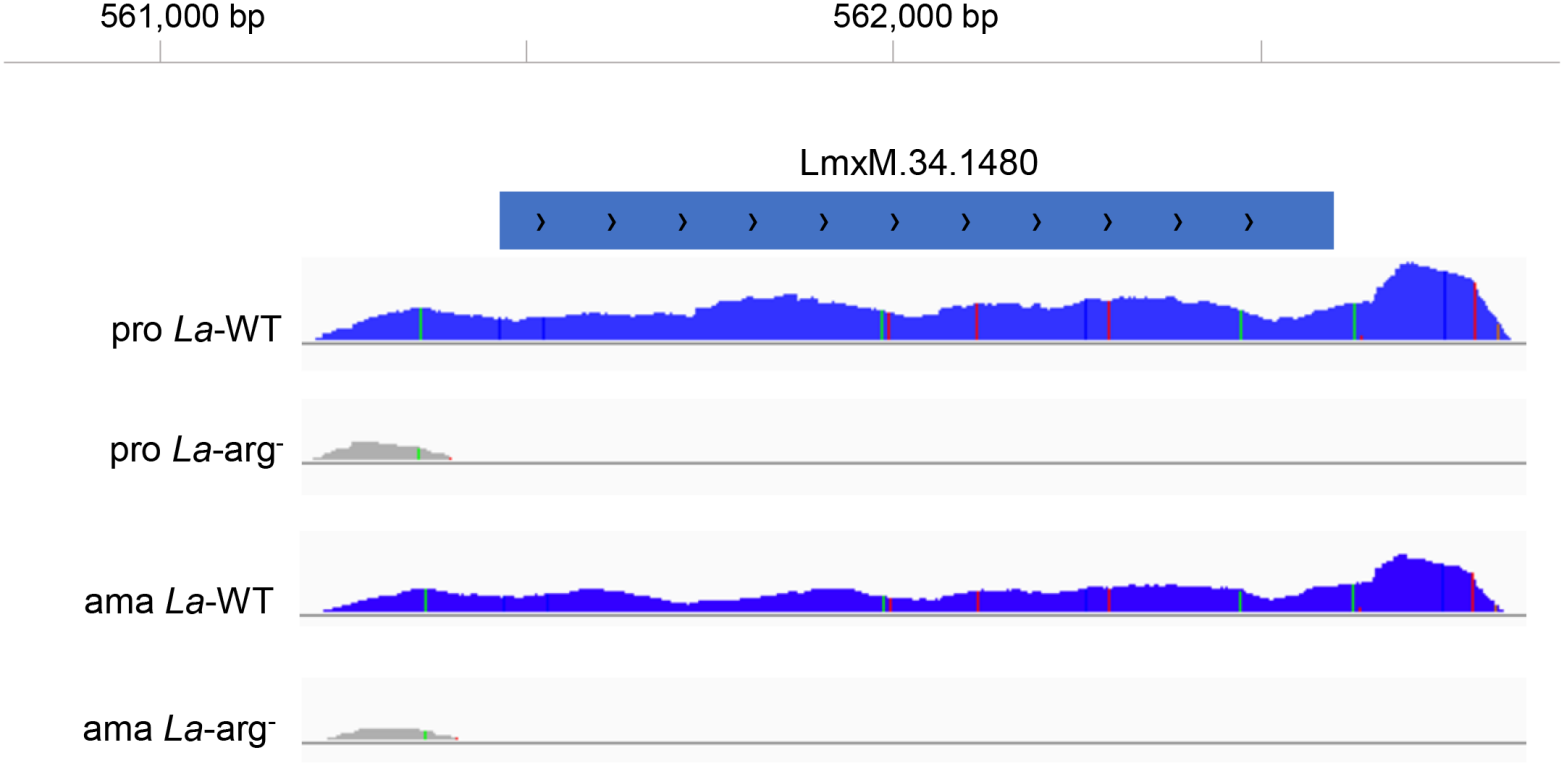
Arginase (LmxM.34.1480) transcript assembling and annotation. Total reads coverage aligned in the region of LmxM.34.1480 gene in the lines *La*-WT and *La*-arg^-^promastigotes (pro) and axenic amastigotes (ama) of the previously annotated *L*. *mexicana* genome. (*La*-WT) *L*. *amazonensis* wild-type, (*La*-arg^-^) *L*. *amazonensis* arginase knockout.

According to Fig 1 we demonstrated the arginase transcript (LmxM.34.1480) assembling with genome coverage in *La*-WT promastigotes and axenic amastigotes. As expected no transcripts were detected in the knockout lines (*La*-arg^-^ promastigotes and axenic amastigotes).

### Identification of differentially expressed genes of *La*-WT and *La*-arg^-^promastigotes and axenic amastigotes

**Fig 2:**
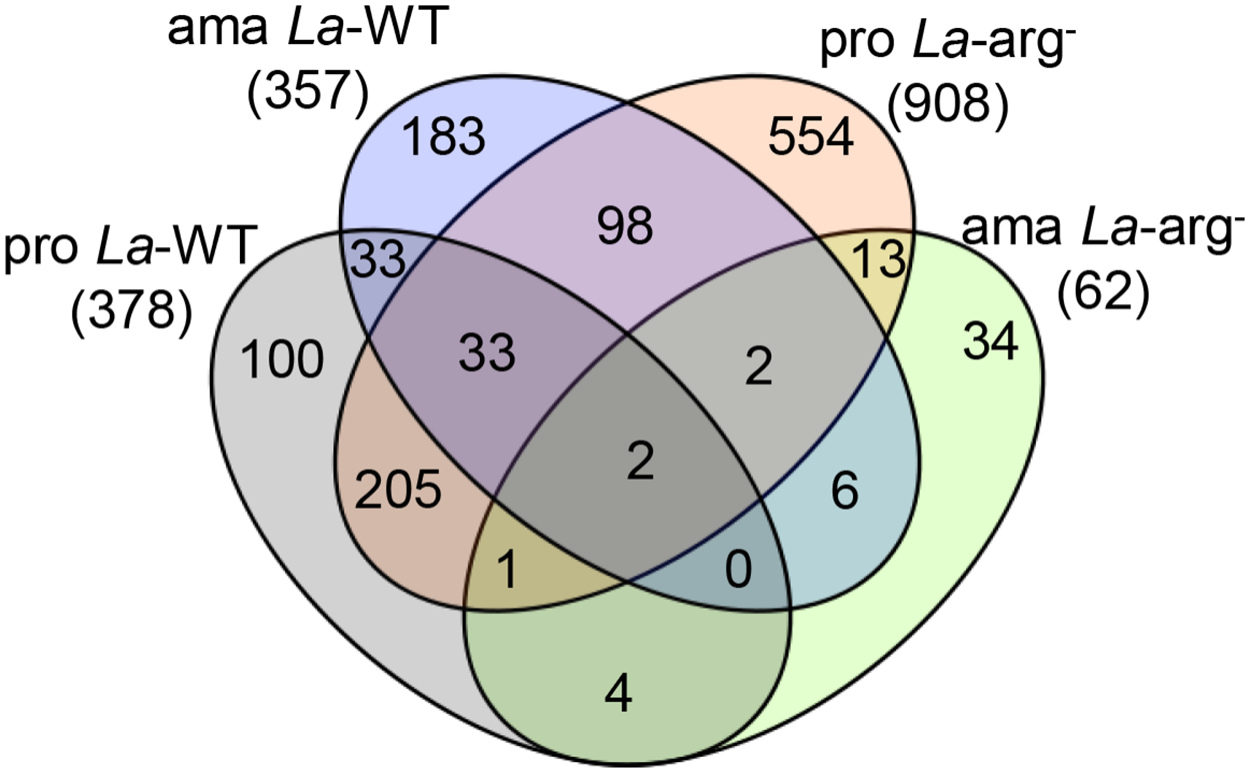
Transcriptional profiling of *La*-WT and *La*-arg^-^ promastigotes and axenic amastigotes. Venn diagram of the 1268 differentially expressed genes, showing the number of genes common and non-common from each sample and the number in the overlap. (pro) promastigote, (ama) axenic amastigote, (*La*-WT) *L*. *amazonensis* wild-type, (*La*-arg^-^) *L*. *amazonensis* arginase knockout.

RNA-seq has been described as an accurate method for quantifying transcript levels [10–12, 28, 29]. Our RNA-seq data revealed that 85% of the genes were constitutively expressed, comparing the gene expression profiles of *La*-WT and *La*-arg^-^ promastigotes and axenic amastigotes. However, among 15% (1268 genes) DE genes, we identified a vast number of genes differentially expressed. Of the total 378 and 357 DE genes in *La*-WT promastigotes and axenic amastigotes, 100 and 183 genes were non-common for each line, respectively. Of the total 908 and 62 DE genes in *La*-arg^-^ promastigotes and axenic amastigotes, 554 genes and 34 genes were non-common for each line, respectively (Fig 2). A direct overlap revealed only 2 transcripts mutually expressed among all samples (Fig 2).

**Fig 3:**
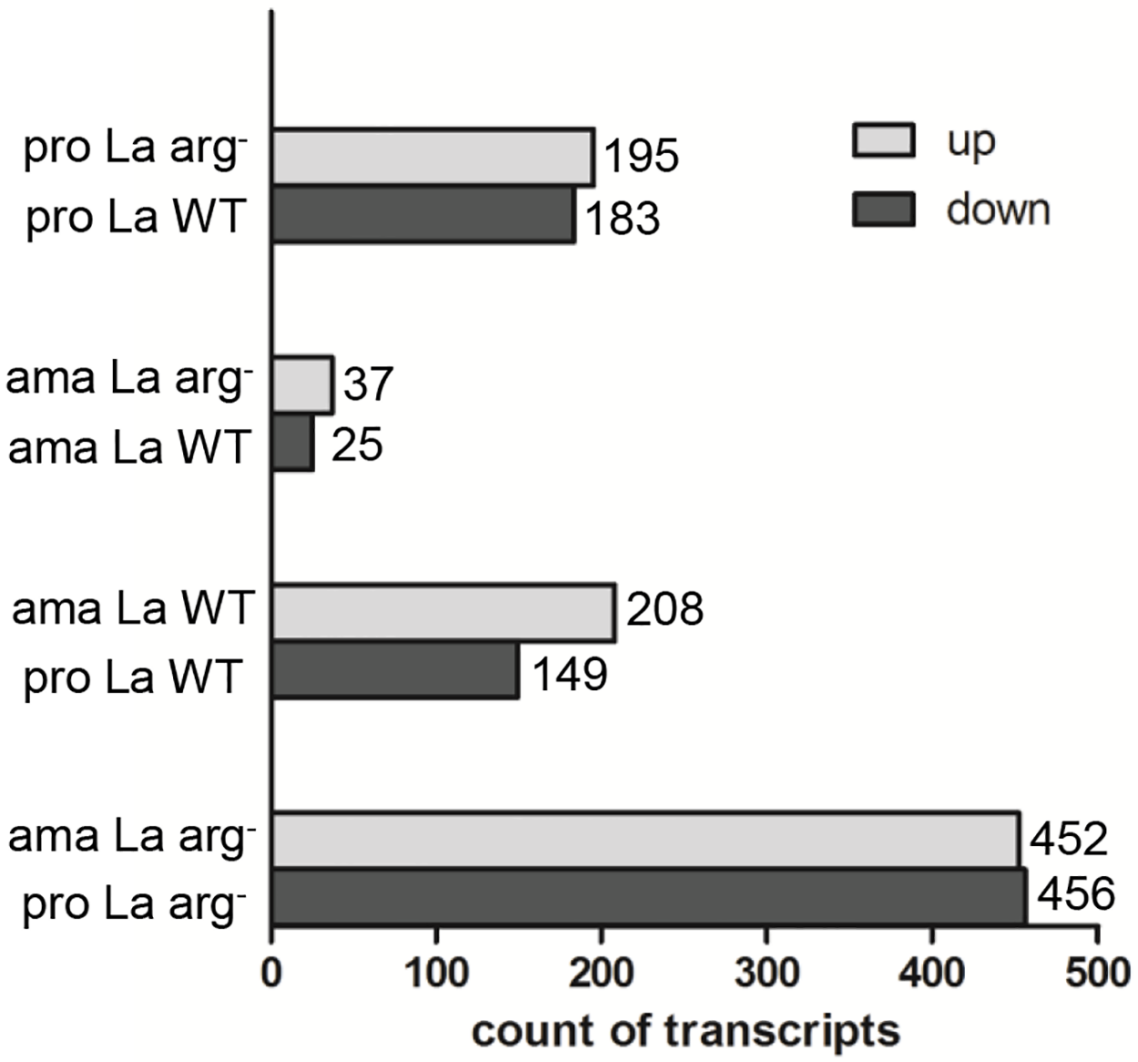
Differential gene expression profiling in *La*-WT and *La*-arg^-^ promastigotes and axenic amastigotes. Count of transcripts up-regulated (light gray) or down-regulated (dark gray) in the comparison of *La*-WT promastigotes vs. *La*-arg^-^promastigotes, *La*-WT axenic amastigotes vs. *La*-arg^-^ axenic amastigotes, *La*-WT promastigotes vs. *La*-WT axenic amastigotes, and *La*-arg^-^ promastigotes vs. *La*-arg^-^axenic amastigotes, considering a fold change ≥ 2 and p value ˂ 0.05. (pro) promastigote, (ama) axenic amastigote, (*La*-WT) *L*. *amazonensis* wild-type, (*La*-arg^-^) *L*. *amazonensis* arginase knockout.

The analyses of the DE genes, limited to those presented fold change ≥ 2 and p ˂ 0.05, revealed 195 genes up-regulated and 183 genes down-regulated in the comparison of *La*-WT promastigote vs. *La*-arg^-^ promastigote, suggesting a significant amount of DE genes which regulation depends on arginase activity. On the other hand, in the comparison of *La*-arg^-^ axenic amastigotes vs. *La*-WT axenic amastigotes, only 37 genes were up-regulated and 25 genes were down-regulated. In addition, in the comparison of *La*-WT axenic amastigotes vs. *La*-WT promastigotes we observed 208 up-regulated and 149 down-regulated genes, indicating a significant amount of DE genes during *La*-WT differentiation. The comparison of *La*-arg^-^ axenic amastigotes vs. *La*-arg^-^ promastigotes led to larger number of DE genes (452 up-regulated and 456 down-regulated genes) what could be explained considering the two variables in this comparison, the absence of arginase activity and the life cycle stage (Fig 3).

The additional characterization of axenic amastigotes transcripts revealed up-regulation of the following amastins: amastin (LmxM.30.0451 and LmxM.30.0452) and amastins-like (LmxM.08.0750, LmxM.08.0760, LmxM.08.0770, LmxM.08.0800, LmxM.08.0850, LmxM.33.0960, LmxM.33.0961, LmxM.33.1560 and LmxM.33.1920) (S2 Fig).

Based on the DE genes analyzed, we generated volcano plots showing the distribution of transcripts by comparing the fold change in the expression (log_2_) of each group with the corresponding adjusted p value (-log_10_) (S3 Fig). We further analyzed the volume plot (S4 Fig) identifying the top 5 transcripts that showed higher expression difference compared to the control according to expression volume (Table 1). Comparing the expression profile of *La*-WT vs. *La*-arg^-^ promastigotes, we identified the following up-regulated transcripts: a conserved hypothetical protein (LmxM.15.1520) and a non-coding RNA (LmxM.23.ncRNA rfamscan: 218578-218718-1); and the following down-regulated transcripts: a putative nucleolar RNA-binding protein (LmxM.07.0990) and two histone H4 proteins (LmxM.06.0010 and LmxM.15.0010). Comparing the expression of *La*-WT vs. *La*-arg^-^ axenic amastigotes, we identified the following up-regulated transcripts: a putative dipeptidyl-peptidase III (LmxM.05.0960), the protein disulfide isomerase (LmxM.06.1050) and a conserved hypothetical protein (LmxM.08.0540); and the following down-regulated transcripts: a conserved hypothetical protein (LmxM.17.0890) and a putative tryparedoxin 1 protein (LmxM.08_29.1160). The comparison of the expression profile of *La*-WT promastigotes vs. axenic amastigotes led to the identification of the following up-regulated transcripts: the tryparedoxin peroxidase (LmxM.15.1160) and a putative ATP-dependent RNA helicase (LmxM.33.2050); and the following down-regulated transcripts: a putative histone H3 (LmxM.10.0970), the histone H4 (LmxM.15.0010) and an unspecific product (LmxM.13.0290partial). Finally, the comparison of the expression profile of *La*-arg^-^ promastigotes vs. axenic amastigotes led to the identification of only down-regulated transcripts: an alpha tubulin (LmxM.13.0280), two beta tubulins (LmxM.32.0792, LmxM.32.0794), an unspecific product (LmxM.13.0300) and a non-coding RNA (LmxM.23.ncRNA rfamscan: 218682-218827-1).

**Table 1:** Top 5 transcripts differentially expressed genes of *La*-WT and *La*-arg^-^ promastigotes and axenic amastigotes.

ID	pro *La*-WT vs. pro *La*-arg^-^ product description	fold change
LmxM.15.1520	hypothetical protein, conserved	3.98
LmxM.07.0990	nucleolar RNA-binding protein, putative	0.37
LmxM.06.0010	histone H4	0.38
Lmx.15.0010	histone H4	0.41
LmxM.23.ncRNA	rfamscan: 218578-218718-1	3.11
ID	ama *La*-WT vs. ama *La*-arg^-^ product description	fold change
LmxM.05.0960	dipeptidyl-peptidase III, putative	2.40
LmxM.06.1050	protein disulfide isomerase	2.35
LmxM.08.0540	hypothetical protein, conserved	2.00
LmxM.17.0890	hypothetical protein, conserved	0.53
LmxM.08_29.1160	tryparedoxin 1, putative (TXN1)	0.47
ID	pro *La*-WT vs. ama *La*-WT product description	fold change
LmxM.15.1160	tryparedoxin peroxidase	2.97
LmxM.33.2050	ATP-dependent RNA helicase, putative	2.00
LmxM.10.0970	histone H3, putative	0.41
LmxM.15.0010	histone H4	0.40
LmxM.13.0290partial	unspecific product	0.37
ID	pro *La*-arg^-^ vs. ama *La*-arg^-^ product description	fold change
LmxM.13.0280	alpha tubulin	0.32
LmxM.32.0792	beta tubulin	0.29
LmxM.13.0300	unspecified product	0.32
LmxM.32.0794	beta tubulin	0.20
LmxM.30.ncRNA	rfamscan: 218682-218827-1	0.27

Top 5 up- and down-regulated transcripts of the total 1,268 previously defined differentially expressed genes between pro *La*-WT vs. pro *La*-arg^-^, ama *La*-WT vs. ama *La*-arg^-^, pro *La*-WT vs. ama *La*-WT, and pro *La*-arg^-^ vs. ama *La*-arg^-^ adjusted for p ˂ 0.05. The list was based on the volume plot of differentially expressed genes, considering log_2_ fold change ≥ 2 and volume obtained from the comparison of the average for each group plotted. X-axis: volume. Y-axis: log_2_ fold change. pro: promastigote, ama: amastigote, *La*-WT: *L*. *amazonensis* wild-type, *La*-arg^-^: *L*. *amazonensis* arginase knockout.

**Fig 4:**
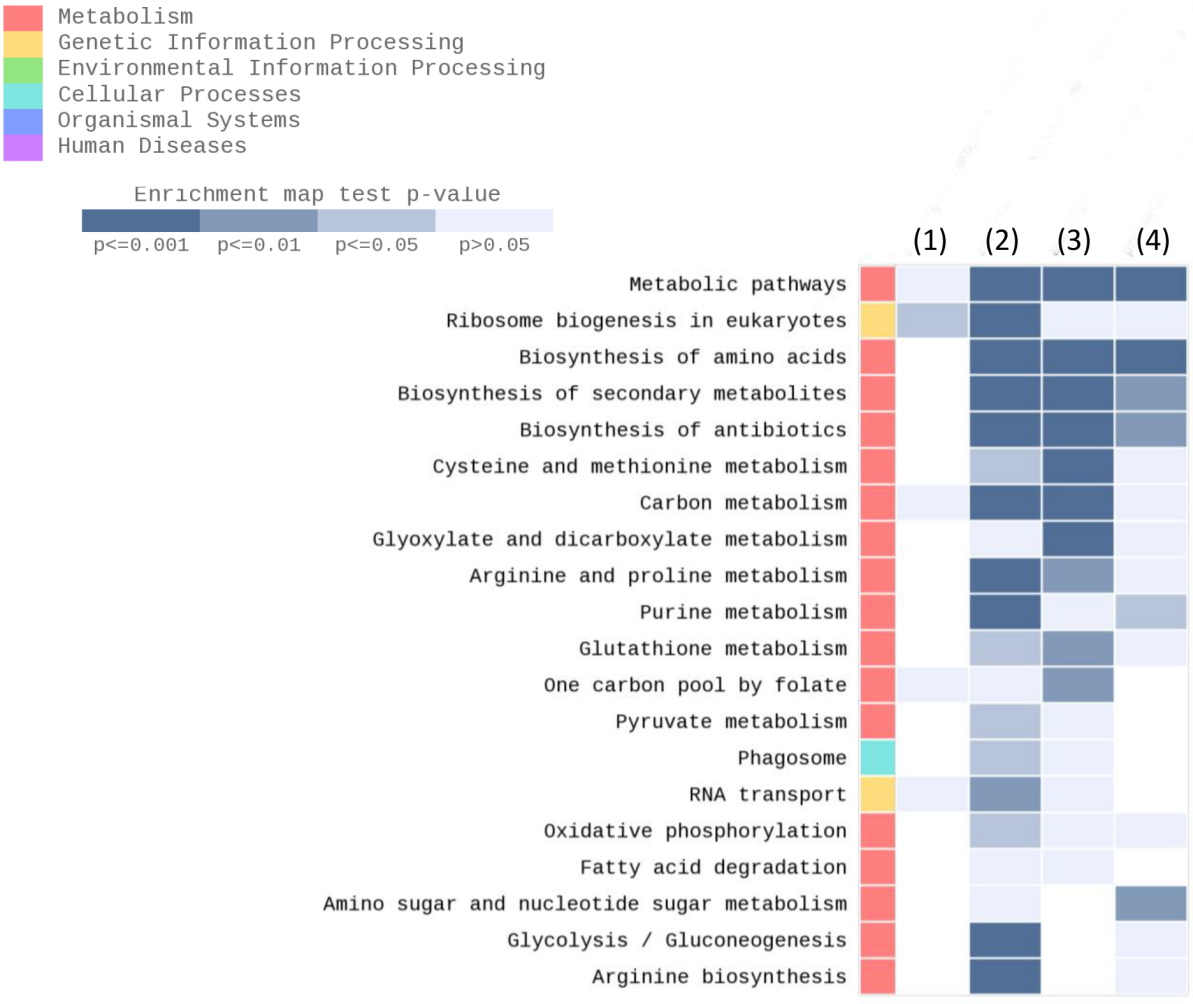
KEEG enrichment analysis showing the list of the top 20 pathways. The heatmap shows the list of the 20 pathways regulated in (1) ama *La*-arg^-^ vs ama *La*-WT, (2) ama *La*-arg^-^ vs pro *La*-arg^-^, (3) ama *La*-WT vs pro *La*-WT and (4) pro *La*-arg^-^ vs pro *La*-WT. The enrichment map is colored by the gradient level of the p value. (pro) promastigote, (ama) axenic amastigote, (*La*-WT) *L*. *amazonensis* wild-type, (*La*-arg^-^) *L*. *amazonensis* arginase knockout.

Furthermore, we performed a KEGG enrichment analysis, which showed a list of the top 20 regulated pathways among all samples. The list includes pathways that can be regulated in the absence of arginase activity, such as the biosynthesis of amino acids, arginine and proline metabolism and arginine biosynthesis (Fig 4 and Table 2).

**Table 2:** KEEG enrichment analyses of *La*-WT and *La*-arg^-^ promastigotes and axenic amastigotes of the following pathways: Biosynthesis of amino acids, arginine and proline metabolism and arginine biosynthesis.

Map ID	Map Name	genes	p value	FDR
01230	Biosynthesis of amino acids	LmxM.06.0370, LmxM.08.29.1570 LmxM.12.0630, LmxM.13.1680 LmxM.14.1320, LmxM.18.0510 LmxM.18.0670, LmxM.26.2710 LmxM.28.1280, LmxM.28.1970 LmxM.28.2370, LmxM.29.3500 LmxM.30.0010, LmxM.34.4750 LmxM.36.1260, LmxM.36.3590	3.60 e^-13^	5.40e^-12^
00330	Arginine and proline metabolism	LmxM.03.0200, LmxM.04.0580 LmxM.12.0280, LmxM.13.1680 LmxM.25.1120, LmxM.26.1610 LmxM.26.2710, LmxM.29.3120 LmxM.34.1480	4.53 e^-07^	4.24 e^-06^
00220	Arginine biosynthesis	LmxM.06.0370, LmxM.07.0270 LmxM.08.29.1570, LmxM.12.0630 LmxM.15.1010, LmxM.23.0260 LmxM.34.1480	9.00 e^-04^	4.00 e^-03^

KEEG enrichment analyses of *La*-WT and *La*-arg^-^ promastigotes and axenic amastigotes highlighting the following pathways: biosynthesis of amino acids, arginine and proline metabolism and arginine biosynthesis. Genes that were differentially expressed are representing, according to p value and False Discovery Rate (FDR).

In this work, we focused on the L-arginine pathway and crossed the obtained data with previous metabolome fingerprints, determined by capillary electrophoresis, also focusing on L-arginine metabolism and the modulation of polyamine metabolism comparing *La*-WT and *La*-arg^-^ promastigotes. Castilho-Martins et al. (2015) observed that the absence of arginase activity led to an increase of L-arginine and citrulline levels, but a decrease of ornithine, proline and putrescine levels. These results confirmed the importance of L-arginine supplying the polyamine pathway in *L*. *amazonensis* and also showed a possible alternative pathway to provide substrates for the pathway in the absence of arginase in the parasite [9]. In fact, to understand why the absence of arginase induced an increase in L-arginine and citrulline levels and a decrease in ornithine, proline and putrescine levels, we analyzed the transcripts levels of specific enzymes involved in these pathways, such as arginase (LmxM.34.1480/EC3.5.3.1), pyrroline 5-carboxylate reductase (LmxM.13.1680/EC1.5.1.2), pyrroline 5-carboxylate dehydrogenase (LmxM.03.0200/EC1.2.1.88), glutamate 5-kinase (LmxM.26.2710/EC2.7.2.11), spermidine synthase (LmxM.04.0580/EC 2.5.1.16), acetylornithine deacetylase (LmxM.07.0270/EC3.5.1.16) and arginosuccinate synthase (LmxM.23.0260/EC6.3.4.5) (Table 3).

Arginase transcripts were not detected in the two knockout lines (*La*-arg^-^ promastigotes and *La*-arg^-^ axenic amastigotes), as expected. Interestingly, a reduced level of arginase was observed in *La*-WT axenic amastigotes, compared to promastigotes. The increase of pyrroline 5-carboxylate reductase transcripts in *La*-arg^-^ compared to *La*-WT promastigotes showed 1.42-fold change. Additionally, an increase of glutamate 5-kinase transcripts was observed in both *La*-WT *and La*-arg^*-*^ axenic amastigotes, compared to promastigotes. On the other hand, we observed decreased transcripts levels of pyrroline 5-carboxylate dehydrogenase, spermidine synthase, acetylornithine deacetylase and arginosuccinate synthase, with 0.76, 0.31, 0.79 and 0.72-fold change, respectively (Table 3).

**Table 3:** Transcripts expression of enzymes involved in the L-arginine pathway in the comparison of *La*-WT and *La*-arg^-^ promastigotes and axenic amastigotes.

Gene ID	EC number	pro *La*-WT vs. pro *La*-arg^*-*^ Product description	fold change
LmxM.34.1480	3.5.3.1	arginase	0.00
LmxM.13.1680	1.5.1.2	pyrroline 5-carboxylate reductase	1.42
LmxM.03.0200	1.2.1.88	pyrroline 5-carboxylate dehydrogenase	0.76
LmxM.04.0580	2.5.1.16	spermidine synthase	0.31
LmxM.07.0270	3.5.1.16	acetylornithine deacetylase	0.79
LmxM.23.0260	6.3.4.5	argininosuccinate synthase	0.72
LmxM.26.2710	2.7.2.11	glutamate 5-kinase	0.80
Gene ID	EC number	pro *La*-WT vs. ama *La-*WT Product description	Fold change
LmxM.34.1480	3.5.3.1	arginase	0.00
LmxM.13.1680	1.5.1.2	pyrroline 5-carboxylate reductase	0.53
LmxM.03.0200	1.2.1.88	pyrroline 5-carboxylate dehydrogenase	1.02
LmxM.04.0580	2.5.1.16	spermidine synthase	0.91
LmxM.07.0270	3.5.1.16	acetylornithine deacetylase	0.88
LmxM.23.0260	6.3.4.5	argininosuccinate synthase	0.50
LmxM.26.2710	2.7.2.11	glutamate 5-kinase	2.58
Gene ID	EC number	ama *La*-WT vs. ama *La-*arg^-^ Product description	Fold change
LmxM.34.1480	3.5.3.1	arginase	0.00
LmxM.13.1680	1.5.1.2	pyrroline 5-carboxylate reductase	0.83
LmxM.03.0200	1.2.1.88	pyrroline 5-carboxylate dehydrogenase	1.22
LmxM.04.0580	2.5.1.16	spermidine synthase	1.02
LmxM.07.0270	3.5.1.16	acetylornithine deacetylase	1.38
LmxM.23.0260	6.3.4.5	argininosuccinate synthase	1.23
LmxM.26.2710	2.7.2.11	glutamate 5-kinase	0.74
Gene ID	EC number	pro *La*-arg^-^ vs. ama *La-*arg^-^ Product description	Fold change
LmxM.34.1480	3.5.3.1	arginase	0.00
LmxM.13.1680	1.5.1.2	pyrroline 5-carboxylate reductase	0.30
LmxM.03.0200	1.2.1.88	pyrroline 5-carboxylate dehydrogenase	1.62
LmxM.04.0580	2.5.1.16	spermidine synthase	2.93
LmxM.07.0270	3.5.1.16	acetylornithine deacetylase	1.54
LmxM.23.0260	6.3.4.5	argininosuccinate synthase	0.86
LmxM.26.2710	2.7.2.11	glutamate 5-kinase	2.40

Comparison of transcripts involved in the arginine pathway of promastigotes (pro) and axenic amastigotes (ama) of *La*-WT and *La*-arg^-^, adjusted for p ˂ 0.05. *La*-WT: *L*. *amazonensis* wild-type, *La*-arg^-^: *L*. *amazonensis* arginase knockout.

**Fig 5:**
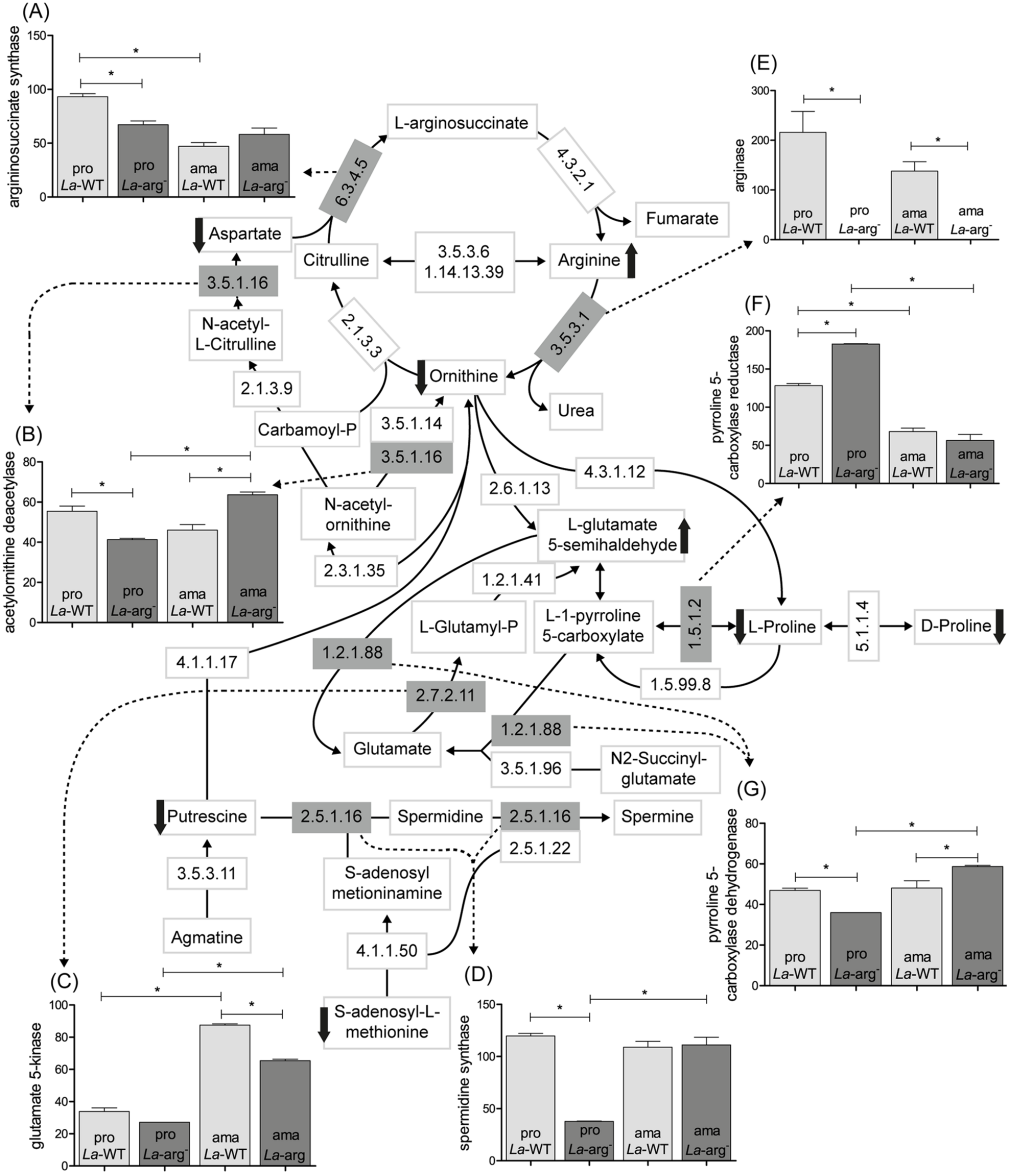
Crossing of transcriptome and metabolome profiles of the arginine pathway, highlighting the enzymes with differential gene expression based on the RNA-seq data. Representation of the arginine pathway and FPKM expression levels highlighting in gray boxes the enzymes with differential gene expression: (A) argininosuccinate synthase (EC6.3.4.5), (B) acetylornithine deacetylase (EC3.5.1.16), (C) glutamate 5-kinase (EC2.7.2.11), (D) spermidine synthase (EC2.5.1.16), (E) arginase (EC3.5.3.1), (F) pyrroline 5-carboxylase reductase (EC1.5.1.2) and (G) pyrroline 5-carbolylate dehydrogenase (EC1.2.1.88). The arrows indicate the increase or decrease levels, according to our RNA-seq data analyses and the metabolome fingerprints, previously described [9]. Each bar is represented from three independent biological replicates. Statistical analyses were performed using *t*-test. (*) p < 0.05.

The crossing of these findings with metabolome data could explain that the increase of L-arginine and citrulline levels could be a consequence of the absence of arginase activity. The increase of L-glutamate levels could be related to the increase of pyrroline 5-carboxylase reductase transcripts. Further, as a consequence of the high consumption of L-glutamate, we observed the decrease of proline that could be related to the decrease of pyrroline 5-carboxylase dehydrogenase transcripts. The decrease of putrescine levels could be related to the decrease of spermidine synthase transcripts. The decrease of ornithine levels could be related to the decrease of acetylornithine deacetylase transcripts. And finally, the decrease of aspartate could be related to the decrease of arginosuccinate synthase transcripts (Fig 5).

Additionally, we performed RT-qPCR validation of some enzymes as shown in S5 Fig.

Here, we described the DE gene profile comparing the expression in promastigotes and axenic amastigotes in the presence or absence of arginase activity. In addition, we performed a correlation analysis with the KEEG arginine pathway, highlighting the regulated enzymes, as previously described in the metabolome work.

## Discussion

L-arginine is an amino acid used as precursor not only for protein synthesis, but also for the synthesis of NO, urea, ornithine, citrulline, creatinine, agmatine, L-glutamate, proline and polyamines [30]. On the other hand, arginase is an enzyme with regulatory roles, modulating L-arginine availability and production of ornithine, a precursor of polyamines, essential for cell replication [30]. Therefore, arginine biosynthesis is an important pathway that not only participates in the regulation of NOS2 parasite killing and arginase-mediated parasite growth, but is also involved in the regulation of the immune system [31, 32].

The infection of murine macrophages with *L*. *amazonensis* showed increased levels of arginase I, La-arginase, arginine transporters (CAT2B and LaAAP3) and miRNA modulation [32]. However, infection with *La*-arg^-^ induces NOS2 expression and the production of NO, causing a lower infection index [8] and blocking miRNA expression [32]. These changes in gene regulation can indicate mechanisms to subvert the defense mechanism developed by the parasite [32, 33].

RNA-seq technology has been used to describe transcriptomic profiles of *L*. *major*, *L*. *mexicana* and *L*. *braziliensis* [10–13, 34]. All of these studies have provided additional knowledge about *Leishmania* biology and the coordinated response of *Leishmania*-infected macrophages in relation to gene regulation at the transcriptional level [12, 13, 29]. RNA-seq data are also helping to revise the previous genome annotation of *L*. *mexicana* [34] and to reconstruct some genomic regions of the *L*. *major* genome that were misassembled [35] in an attempt to improve the current genome and gene annotations.

In this work, using the RNA-seq approach, we described the transcriptional profiling of *L*. *amazonensis*, compared to the phylogenetically close *L*. *mexicana* genome, since *L*. *amazonensis* genome is not completely annotated [27, 36, 37]. We obtained RNA-seq data from *La*-WT and *La*-arg^-^ promastigotes and axenic amastigotes allowing the comparison of transcript abundance from different life cycle stages in the presence or absence of arginase, an important enzyme of the parasite´s polyamines synthesis.

From the 8253 transcripts identified in *La*-WT and *La*-arg^-^ promastigotes and axenic amastigotes, 60% of them were identified as hypothetical proteins and 443 were identified as novel transcripts, that did not correspond to any previously annotated genes. Although we obtained less transcripts than previously predicted for *L. mexicana [34]*, we could assure that the transcripts identified fulfill confidence coverage of the RNA-seq data described in this work. Recent studies have been showing the importance of characterizing a hypothetical protein not only by functional genomics, but also according to its general biological features, allowing the acquisition of new knowledge about signaling pathways, metabolism, stress response, drug resistance and in the identification of new therapeutic targets [38]. The identification of novel transcripts has been improving the accuracy of the *L*. *amazonensis* genome [34, 35]. The finding of novel transcripts can suggest that the gene content of this organism may be higher than previously determined because the majority of novel transcripts contain open reading frames shorter than coding sequencing of genes in the current genome annotation. Another explanation could point to a novel genome organization and processing [29, 34, 39].

*L*. *major* chromosomes are organized as large clusters of genes or open reading frames in the same 5´- 3´ direction on the same DNA strand [40]. Each cluster of genes is processed by a transcription initiation site in a polycistronic transcription [41]. Individual mRNA transcripts are then processed, co-transcriptionally, by 5´RNA splicing and 3´polyadenylation. Therefore, without a gene specific promoter, the entire chromosome is constitutively transcribed [41].

The analysis of the transcriptome revealed 85% of genes were constitutively expressed in promastigotes and axenic amastigotes of *L*. *amazonensis*. The *Leishmania* genome has been described as constitutively expressed, indicating that the parasite is adapted for survival and replication in the sand-fly vector or macrophage host, using an appropriate set of genes/proteins for different environments [40]. Altogether, these results support the previous hypothesis that the *Leishmania* genome is mostly constitutively expressed.

Interestingly, among the 1268 DE genes identified in this work, most was detected in *La*-arg^-^ promastigotes (908 genes). *La*-arg^-^ do not use L-arginine to produce ornithine because arginase activity is absent in this parasite line that requires polyamine supplementation for survival and replication [8]. *La*-arg^-^ also presented a higher concentration of L-arginine in the cytoplasm in relation to the *La*-WT promastigote, probably because the parasite can sense the L-arginine pool, that leads to the regulation of L-arginine transporter expression and L-arginine uptake [42]. The absence of arginase can induce the parasite to regulate many genes involved in the arginine pathway. Therefore, the null mutant *La*-arg^-^ was previously characterized and the essentiality of arginase in *Leishmania in vitro* growth was demonstrated with the requirement of putrescine supplementation [8]. In contrast, the arginase add-back mutant line (*La*-arg^-^/+ARG) restored arginase expression, growth and infectivity *in vivo* [8] and *in vitro* [32] assays. So, we focused on arginine pathway comparing our RNA-seq data with metabolome fingerprints, previously described by our group [9].

The first transcript analyzed was arginase (EC3.5.3.1) and, as expected, no transcript was observed in both life cycle stages of the arginase knockout line (*La*-arg^-^ promastigotes and *La*-arg^-^ axenic amastigotes), reinforcing the efficiency of the knockout methodology used [8] and indicating that the profile is maintained after amastigote differentiation. The metabolomic analysis of *La*-arg^-^ promastigotes showed that absence of arginase causes an increase in L-arginine and citrulline levels, and the decrease in ornithine, putrescine and proline levels, indicating an alternative pathway to surpass the lack of this enzyme [9, 42]. Citrulline could be metabolized by arginine deiminase (EC3.5.3.6) or oxidoreductases (EC1.14.13.39/EC1.14.13.165). Arginine deiminase acts on carbon-nitrogen bonds [43]. Oxidoreductases act on paired donors, with incorporation or reduction of molecular oxygen on arginine biosynthesis [44]. It is interesting to note that similar transcript levels of this oxidoreductase were observed in *La*-WT and *La*-arg^-^ promastigote and axenic amastigotes.

Furthermore, the decrease in the levels of aspartate, ornithine, proline and putrescine indicates that this pathway can be used as an alternative pathway due to the differential expression of argininosuccinate synthase (EC6.3.4.5), acetylornithine deacetylase (EC3.5.1.16), pyrroline-5-carboxylate reductase (EC1.5.1.2), pyrroline-5-carboxylase dehydrogenase (EC1.2.1.88) and glutamate 5-kinase (EC2.7.2.11). The decrease in aspartate may be due to its conversion to L-arginine succinate by argininosuccinate synthase. The decrease in ornithine could be due to the absence of L-arginine conversion in ornithine by arginase and/or the acetylornithine deacetylase consumption.

Glutamate 5-kinase, which is also involved in glutamate metabolism, was not differentially expressed between *La*-WT and *La*-arg^-^ promastigotes. This could be explained by the maintenance from the substrates L-glutamate to glutamyl-P [9]. Interestingly, increased transcript levels of glutamate 5-kinase in both *La*-WT and *La*-arg^-^ axenic amastigotes were observed. The glutamate metabolism was previously described to be involved in the differentiation of *Trypanosoma cruzi* from epimastigotes to metaclyclic trypomastigotes [45, 46]. The L-glutamate levels were increased in L-arginine deprivation, indicating a role of L-glutamate in L-arginine metabolism to supply the absence of L-arginine uptake [9]. In addition, it was described that, as alanine, L-glutamate has a key role in the cell physiology of *Leishmania* [47].

Other enzymes involved in the arginine pathway were not described in this work since they did not appear to be regulated in the previous metabolome analysis.

In the list of top 5 transcripts differentially expressed based on the volume plot, none of the most regulated transcripts was related to the arginine pathway. However, it presented interesting DE genes. Three conserved hypothetical proteins (LmxM.15.1520, LmxM.08.0540 and LmxM.17.0890) were identified and their characterization is important not only for functional genomics but also to improve the knowledge about signaling pathways, metabolism, the stress response, drug resistance, as well as for the identification of new therapeutic targets [38]. In addition, the identification of histone H3 and H4 (LmxM.06.0010, LmxM.15.0010 and LmxM.10.0970) in the comparisons with *La*-WT (pro *La*-WT vs pro *La*-arg^-^, and pro *La*-WT vs ama *La*-WT) is indicative that histones play a central role in transcription regulation, DNA repair, DNA replication and chromosomal stability [40, 48, 49] in *Leishmania* life cycle, even in the absence of arginase activity. RNA-binding protein (RBP) appeared down-regulated in the comparison pro *La*-WT vs pro *La*-arg^-^. RBPs have been described as regulatory elements controlling the expression of genes, involving changes in mRNA stability and/or translational control. The shift from transcriptional to post-transcriptional control in trypanosomatids appears to be due to a different arrangement of protein coding genes. In addition to this specialized arrangement, the genes lack canonical promoters [40, 50, 51]. Currently, RBPs have been reported in *Leishmania* [52–54] and can elucidate gene expression regulation.

Dipeptidyl-peptidase III (DCP) (LmxM.05.0960) appeared up-regulated in the comparison of *La*-WT vs. *La*-arg^-^axenic amastigotes. DCP belongs to the mono-zinc peptidase family. Peptidases of parasitic protozoa have been suggested as novel virulence factors, potential drug targets and vaccine candidates [55]. Previously, by microarray analyses, DCP was shown to be up-regulated in *L*. *donovani* amastigotes compared to promastigotes due to its correlation with an increase in total protease activity [56]. Thus, DCP may have a role in parasite differentiation related to nutrition and pathogenesis [56]. Similar to DCP, the protein disulfide isomerase (PDI) (LmxM.06.1050) appeared up-regulated in *La*-arg^-^ axenic amastigotes compared to *La*-WT. PDI, a redox chaperone, has been primarily characterized with virulent and immunogenic potential [57, 58]. Achour et al. (2002) suggested that *Leishmania* promastigote growth might be due to optimal protein folding as a result of the increased secretion of PDI at the surface of the parasite [58]. Later, Amit et al. (2014) showed that alanine, an inhibitor of PDI activity, caused damage to the parasite mainly in axenic amastigote forms [57].

Tryparedoxin 1 (TXN1) (LmxM.08_29.1160) was down regulated in *La*-arg^-^ axenic amastigotes compared to *La*-WT. TXN1 is part of the trypanothione and trypanothione reductase pathway to regulate oxidative stress [59]. The polyamine pathway can be considered metabolically important for survival and infectivity in trypanosomatids [4, 6, 60]. On the other hand, tryparedoxin peroxidase (TXNPx) (LmxM.15.1160) was up-regulated in *La*-WT axenic amastigotes compared to *La*-WT promastigotes, corroborating the results of previous studies demonstrating that this increase is necessary for detoxification of peroxides and resistance to NO in *L*. *donovani*, which did not show the same profile in the absence of arginase [61–63].

RNA helicases are central players in RNA biology and function. Similar to other eukaryotes, many biological functions have been attributed to trypanosomatid RNA helicases, including RNA degradation, translation regulation and RNA editing [64–66]. We identified that an ATP-dependent RNA helicase (LmxM.33.2050) was up-regulated in *La*-WT promastigotes compared to *La*-WT axenic amastigotes indicating a rgene regulation in *Leishmania* differentiation.

Interestingly, the comparison of the expression profile of *La*-arg^-^promastigotes vs *La*-arg^-^ axenic amastigotes showed only down-regulated genes: an α-tubulin (LmxM.13.0280), two β-tubulins (LmxM.32.0792 and LmxM.32.0794), an unspecified product (LmxM.13.0300) and a ncRNA (LmxM.30.ncRNA rfamscan: 218682-218827-1). α-tubulin is a highly conserved protein that interacts with β-tubulin, forming an α/β-tubulin heterodimer, a key to the formation of the eukaryotic cytoskeleton, which is responsible for cell shape and it is involved in many essential processes, including cell division and ciliary and flagellar motility [67, 68]. Altogether, these findings of down-regulated genes could be indicative of cytoskeleton reorganization dependent of the absence of arginase activity.

## Conclusions

The transcriptional profiling of *L*. *amazonensis* reinforces the capacity of the parasite to fine-tune gene expression regulation to adapt to changes in the environment during promastigote and amastigote differentiation. It is interesting to note that although gene expression regulation in *Leishmania* is considered to occur at post-transcriptional levels, we observed a correlation between the transcriptomic and metabolomics data, focused on the L-arginine pathway. Additionally, the use of the arginase knockout parasite reinforced the importance of this enzyme and provided additional insights into the coordination of gene expression and parasite development and infectivity.

## Supporting information

S1 Fig *La*-WT and *La*-arg^-^ axenic amastigotes growth profile and BMDMs infection index. Axenic amastigotes were obtained from promastigote culture and differentiated after 48 h through the new conditions in medium, pH 5.5 at 34°C. Axenic amastigotes growth profile and the BMDMs infectivity were evaluated. **(A)** The growth curve of axenic amastigotes of *L*. *amazonensis* wild-type (*La*-WT) and *L*. *amazonensis* arginase knockout (*La*-arg^-^). The initial inoculum was 5x10^6^ cells/mL from promastigotes forms in stationary growth phase. Axenic amastigotes were counted in a Neubauer chamber every 24 h for 10 days. The percentage of cellular growth is represented from three independent biological replicates. **(B)** The infection index of BMDMs infected with *La*-WT and *La*-arg^-^ axenic amastigotes after 24, 48 and 72 h. Bars represent the mean ± standard deviation of three independent biological replicates, calculated by counting 200 Panoptic-stained cells. The infection index was determined by multiplying the percentage of infected macrophages by the mean of the number of parasites per infected cell. Statistical analyses were performed using a *t-*test. (*) p ˂ 0.05, compared to *La*-WT.
(TIF)

S2 Fig Differential gene expression based on the amastin FPKMs expression levels of *La*-WT and *La*-arg^-^ promastigotes and axenic amastigotes. Each bar is represented from three independent biological replicates. Statistical analyses were performed using a *t-*test. (*) p ˂ 0.05. **(A)** amastin-like (LmxM.08.0750) FPKM expression levels. **(B)** amastin-like (LmxM.08.0760) FPKM expression levels. **(C)** amastin-like (LmxM.08.0770) FPKM expression levels. **(D)** amastin-like (LmxM.08.0800) FPKM expression levels. **(E)** amastin-like (LmxM.08.0850) FPKM expression levels. **(F)** amastin (LmxM.30.0452) FPKM expression levels. **(G)** amastin (LmxM.30.0451) FPKM expression levels. **(H)** amastin-like (LmxM.33.0960) FPKM expression levels. **(I)** amastin-like (LmxM.33.0961) FPKM expression levels. **(J)** amastin-like (LmxM.33.1560) FPKM expression levels. **(K)** amastin-like (LmxM.33.1920) FPKM expression levels. (pro) promastigote, (ama) axenic amastigote, (*La*-WT) *L*. *amazonensis* wild-type, (*La*-arg^-^) *L*. *amazonensis* arginase knockout.
(TIF)

S3 Fig Volcano plot of the differentially expressed genes of *La*-WT and *La*-arg^-^ promastigotes and axenic amastigotes. Volcano plot of the comparisons, considering a fold change ≥ 2 and p value ˂ 0.05. Genes significantly up-regulated (yellow dots) are located at the upper right square of each graph (positive log fold value). Genes significantly down-regulated (blue dots) are located at the upper left square of each graph (negative log fold value). **(A)** Volcano plot from the comparison of pro *La*-WT and pro *La*-arg^-^. **(B)** Volcano plot from the comparison of ama *La*-WT and ama *La*-arg^-^. **(C)** Volcano plot from the comparison of pro *La*-WT and ama *La*-WT. **(D)** Volcano plot from the comparison of pro *La*-arg^-^ and ama *La*-arg^-^. Log_2_ fold change and p value were obtained from the comparison of the average for each group plotted. X-axis: log_2_ fold change. Y-axis: -log_10_ p value. (pro) promastigote, (ama) axenic amastigote, (*La*-WT) *L*. *amazonensis* wild-type, (*La*-arg^-^) *L*. *amazonensis* arginase knockout.
(TIF)

S4 Fig Volume plot of the differentially expressed genes of *La*-WT and *La*-arg^-^ promastigotes and axenic amastigotes. Volume plot of the comparisons, considering a log_2_ fold change ≥ 2 and mean of normalized gene volume. Genes significantly up- and down-regulated (red dots). **(A)** Volume plot from the comparison of pro *La*-WT and pro *La*-arg^-^. **(B)** Volume plot from the comparison of ama *La*-WT and ama *La*-arg^-^. **(C)** Volume plot from the comparison of pro *La*-WT and ama *La*-WT. **(D)** Volume plot from the comparison of pro *La*-arg^-^ and ama *La*-arg^-^. (pro) promastigote, (ama) axenic amastigote, (*La*-WT) *L*. *amazonensis* wild-type, (*La*-arg^-^) *L*. *amazonensis* arginase knockout.
(TIF)

S5 Fig RT-qPCR validation of glutamate 5-kinase, spermidine synthase, arginase, pyrroline 5-carboxylase dehydrogenase and amastin-like expression. mRNA expression of the following enzymes (A) glutamate 5-kinase (LmxM.26.2710/EC2.7.2.11), (B) spermidine synthase (LmxM.04.0580/EC 2.5.1.16), (C) arginase (LmxM.34.1480/EC3.5.3.1), (D) pyrroline 5-carboxylase dehydrogenase (LmxM.03.0200/EC1.2.1.88), (E) amastin-like (LmxM.33.0960) and (F) amastin-like (LmxM.08.0760) in *L*. *amazonensis* wild type (*La*-WT) and *L*. *amazonensis* arginase knockout (*La*-arg^-^) promastigotes (pro) and axenic amastigotes (ama). Data were based on quantification of the target and were normalized by *gapdh* expression. The values are the mean ± SEM of three independent biological replicates.
(TIF)

S1 Table Transcriptomic profiling from *La*-WT and *La*-arg^-^ promastigotes and axenic amastigotes. (PDF)

S2 Table List of transcripts, hypothetical proteins and novel transcripts identified in *La*-WT and *La*-arg^-^ promastigotes and axenic amastigotes transcriptomic profiling. (XLSX)
