# Supplementary figures and images for: RNA-seq transcriptional profiling of Leishmania amazonensis reveals an arginase-dependent gene expression regulation
Source: PLoS Negl Trop Dis. 2017 Oct 27;11(10):e0006026. doi: 10.1371/journal.pntd.0006026 (PMC5678721; doi:10.1371/journal.pntd.0006026)

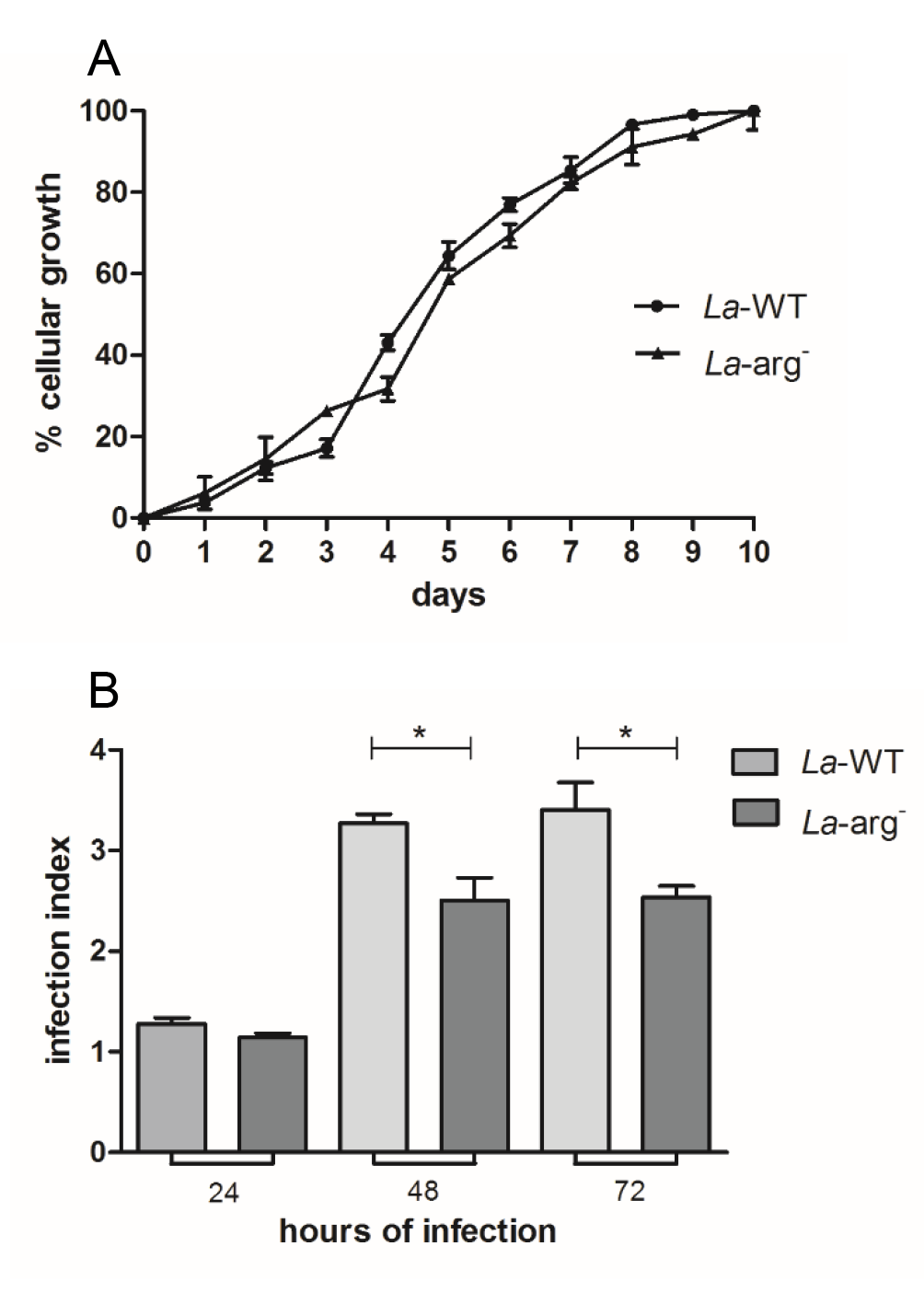

Supplement: S1 Fig — Axenic amastigotes were obtained from promastigote culture and differentiated after 48 h through the new conditions in medium, pH 5.5 at 34°C. Axenic amastigotes growth profile and the BMDMs infectivity were evaluated. (A) The growth curve of axenic amastigotes of L. amazonensis wild-type (La-WT) and L. amazonensis arginase knockout (La-arg-). The initial inoculum was 5x10^6^ cells/mL from promastigotes forms in stationary growth phase. Axenic amastigotes were counted in a Neubauer chamber every 24 h for 10 days. The percentage of cellular growth is represented from three independent biological replicates. (B) The infection index of BMDMs infected with La-WT and La-arg- axenic amastigotes after 24, 48 and 72 h. Bars represent the mean ± standard deviation of three independent biological replicates, calculated by counting 200 Panoptic-stained cells. The infection index was determined by multiplying the percentage of infected macrophages by the mean of the number of parasites per infected cell. Statistical analyses were performed using a t-test. (*) p ˂ 0.05, compared to La-WT. (TIF) [file pntd.0006026.s001.tif]

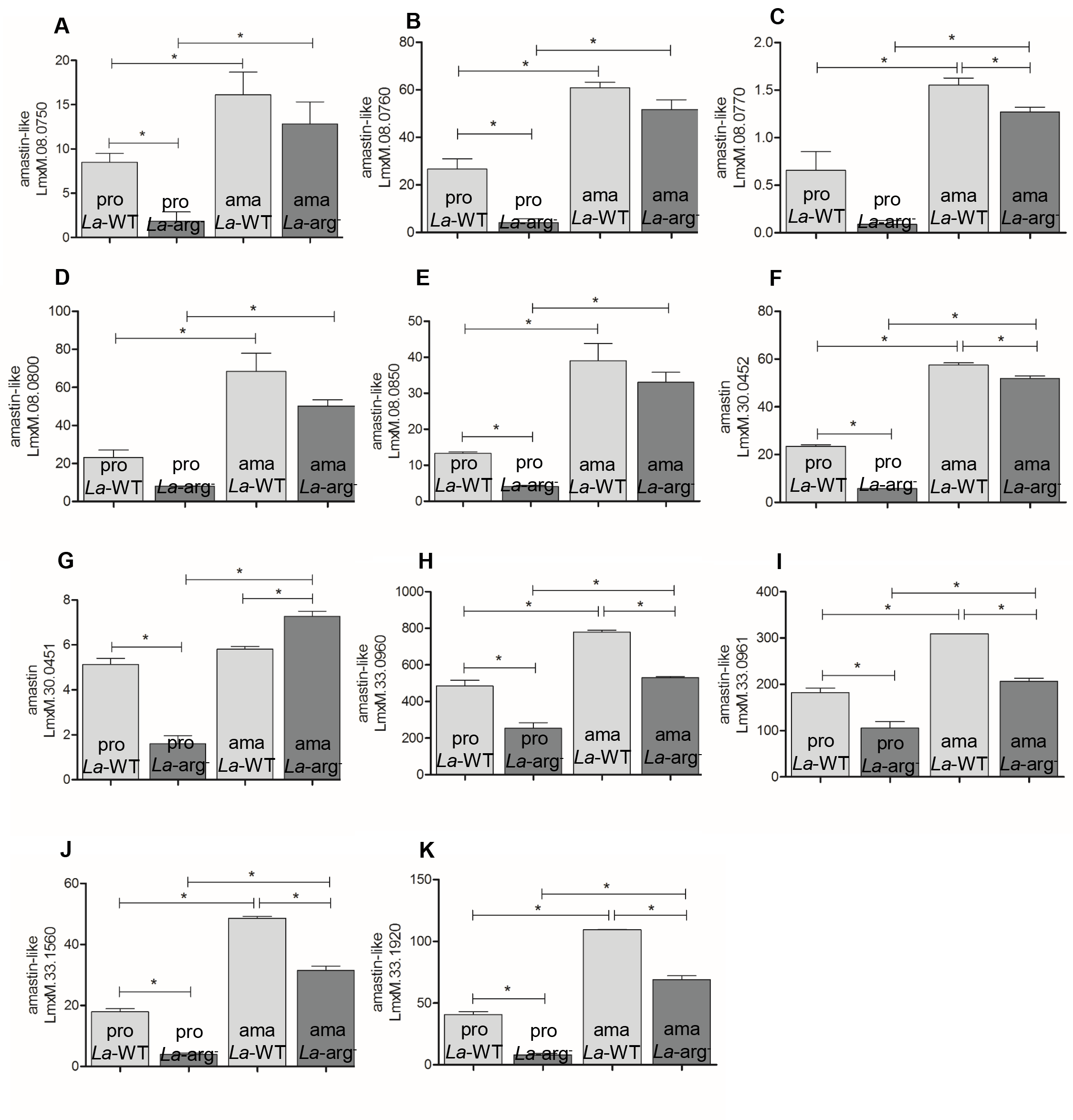

Supplement: S2 Fig — Each bar is represented from three independent biological replicates. Statistical analyses were performed using a t-test. (*) p ˂ 0.05. (A) amastin-like (LmxM.08.0750) FPKM expression levels. (B) amastin-like (LmxM.08.0760) FPKM expression levels. (C) amastin-like (LmxM.08.0770) FPKM expression levels. (D) amastin-like (LmxM.08.0800) FPKM expression levels. (E) amastin-like (LmxM.08.0850) FPKM expression levels. (F) amastin (LmxM.30.0452) FPKM expression levels. (G) amastin (LmxM.30.0451) FPKM expression levels. (H) amastin-like (LmxM.33.0960) FPKM expression levels. (I) amastin-like (LmxM.33.0961) FPKM expression levels. (J) amastin-like (LmxM.33.1560) FPKM expression levels. (K) amastin-like (LmxM.33.1920) FPKM expression levels. (pro) promastigote, (ama) axenic amastigote, (La-WT) L. amazonensis wild-type, (La-arg-) L. amazonensis arginase knockout. (TIF) [file pntd.0006026.s002.tif]

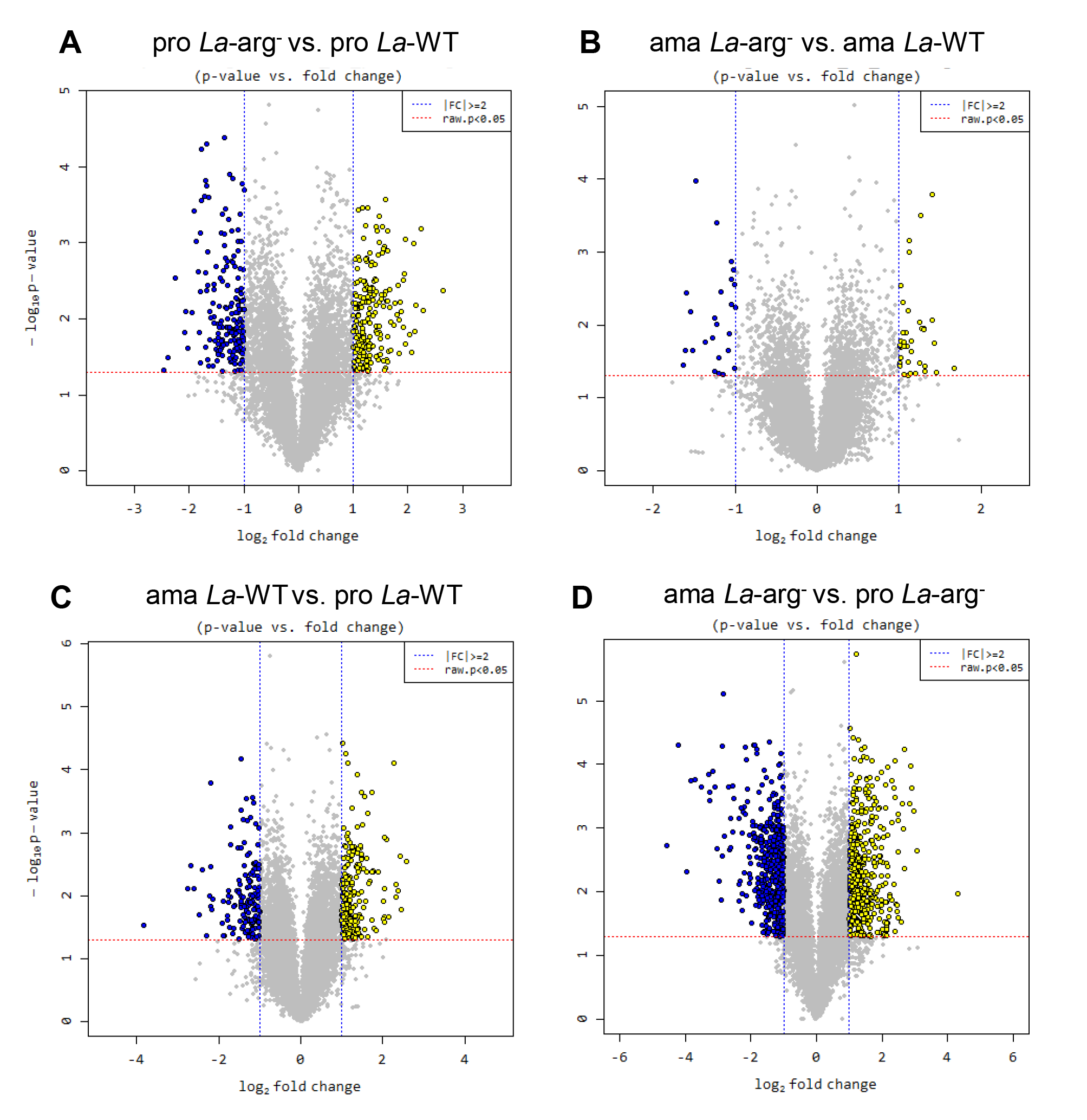

Supplement: S3 Fig — Volcano plot of the comparisons, considering a fold change ≥ 2 and p value ˂ 0.05. Genes significantly up-regulated (yellow dots) are located at the upper right square of each graph (positive log fold value). Genes significantly down-regulated (blue dots) are located at the upper left square of each graph (negative log fold value). (A) Volcano plot from the comparison of pro La-WT and pro La-arg-. (B) Volcano plot from the comparison of ama La-WT and ama La-arg-. (C) Volcano plot from the comparison of pro La-WT and ama La-WT. (D) Volcano plot from the comparison of pro La-arg- and ama La-arg-. Log2 fold change and p value were obtained from the comparison of the average for each group plotted. X-axis: log2 fold change. Y-axis: -log10 p value. (pro) promastigote, (ama) axenic amastigote, (La-WT) L. amazonensis wild-type, (La-arg-) L. amazonensis arginase knockout. (TIF) [file pntd.0006026.s003.tif]

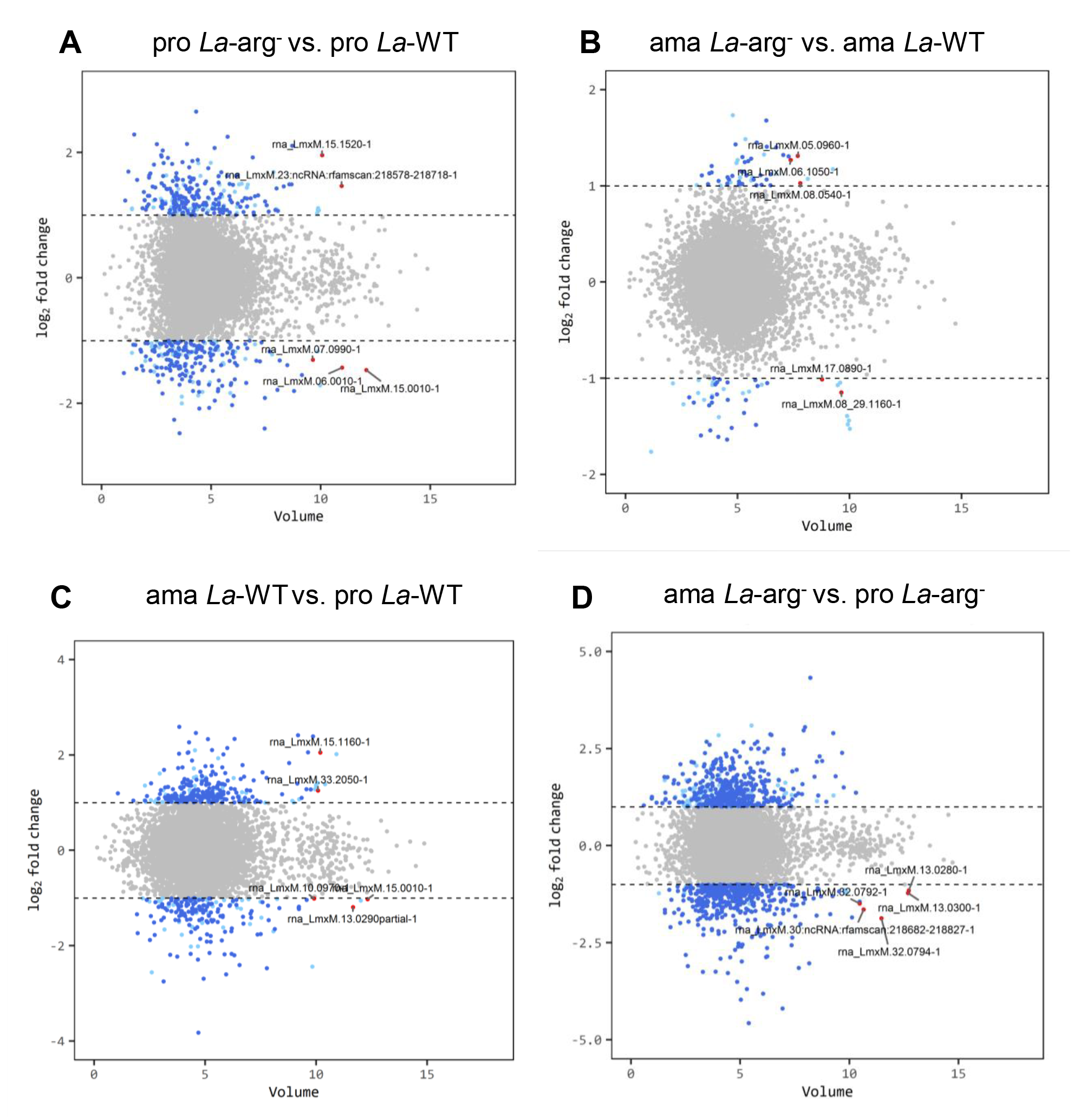

Supplement: S4 Fig — Volume plot of the comparisons, considering a log2 fold change ≥ 2 and mean of normalized gene volume. Genes significantly up- and down-regulated (red dots). (A) Volume plot from the comparison of pro La-WT and pro La-arg-. (B) Volume plot from the comparison of ama La-WT and ama La-arg-. (C) Volume plot from the comparison of pro La-WT and ama La-WT. (D) Volume plot from the comparison of pro La-arg- and ama La-arg-. (pro) promastigote, (ama) axenic amastigote, (La-WT) L. amazonensis wild-type, (La-arg-) L. amazonensis arginase knockout. (TIF) [file pntd.0006026.s004.tif]

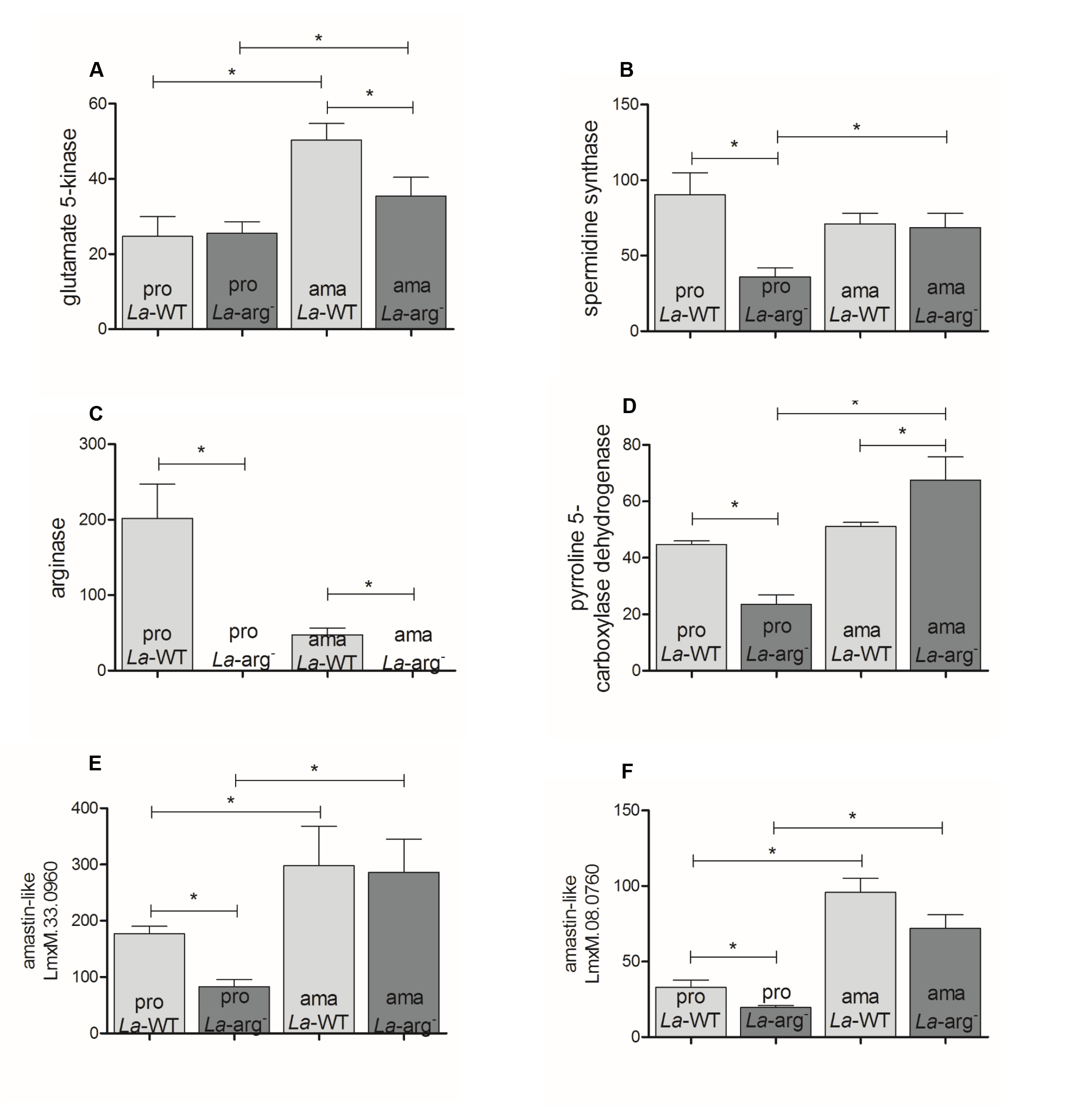

Supplement: S5 Fig — mRNA expression of the following enzymes (A) glutamate 5-kinase (LmxM.26.2710/EC2.7.2.11), (B) spermidine synthase (LmxM.04.0580/EC 2.5.1.16), (C) arginase (LmxM.34.1480/EC3.5.3.1), (D) pyrroline 5-carboxylase dehydrogenase (LmxM.03.0200/EC1.2.1.88), (E) amastin-like (LmxM.33.0960) and (F) amastin-like (LmxM.08.0760) in L. amazonensis wild type (La-WT) and L. amazonensis arginase knockout (La-arg-) promastigotes (pro) and axenic amastigotes (ama). Data were based on quantification of the target and were normalized by gapdh expression. The values are the mean ± SEM of three independent biological replicates. (TIF) [file pntd.0006026.s005.tif]
