# Supplementary material for: RNA-seq transcriptional profiling of Leishmania amazonensis reveals an arginase-dependent gene expression regulation
Source: PLoS Negl Trop Dis. 2017 Oct 27;11(10):e0006026. doi: 10.1371/journal.pntd.0006026 (PMC5678721; doi:10.1371/journal.pntd.0006026)
Supplement: S1 Table — (PDF) [file pntd.0006026.s006.pdf]

S1 Table. Transcriptomic profiling from *La*-WT and *La*-arg⁻ promastigotes and axenic amastigotes

sample	total reads bases	total reads	GC (%)	Q30 (%)
pro *La*-WT	3,649,601,083	30,188,300	58.17	94.83
pro *La*-arg$^-$	4,265,625,299	35,346,610	57.06	95.04
ama *La*-WT	1,437,773,830	11,838,303	57.41	94.98
ama *La*-arg$^-$	23,227,835	5,811,333	58.11	94.42

Total reads bases: total number of bases sequenced
Total reads: total number of reads
GC (%): GC content
Q30 (%): ratio of reads with Phred quality score over 30
(pro) promastigote, (ama) axenic amastigote, (*La*-WT) *L. amazonensis* wild-type, (*La*-arg$^-$) *L. amazonensis* arginase knockout.
